# Decoding spectrotemporal features of overt and covert speech from the human cortex

**DOI:** 10.3389/fneng.2014.00014

**Published:** 2014-05-27

**Authors:** Stéphanie Martin, Peter Brunner, Chris Holdgraf, Hans-Jochen Heinze, Nathan E. Crone, Jochem Rieger, Gerwin Schalk, Robert T. Knight, Brian N. Pasley

**Affiliations:** ^1^Helen Wills Neuroscience Institute, University of CaliforniaBerkeley, CA, USA; ^2^Department of Bioengineering, École Polytechnique Fédérale de LausanneLausanne, Switzerland; ^3^New York State Department of Health, Wadsworth CenterAlbany, NY, USA; ^4^Department of Neurology, Albany Medical CollegeAlbany, NY, USA; ^5^Department of Neurology, Otto-von-Guericke-UniversitatMagdeburg, Germany; ^6^Department of Neurology, Johns Hopkins University School of MedicineBaltimore, MD, USA; ^7^Applied Neurocognitive Psychology, Carl-von-Ossietzky UniversityOldenburg, Germany; ^8^Department of Psychology, University of CaliforniaBerkeley, CA, USA

**Keywords:** electrocorticography, speech production, covert speech, decoding model, pattern recognition

## Abstract

Auditory perception and auditory imagery have been shown to activate overlapping brain regions. We hypothesized that these phenomena also share a common underlying neural representation. To assess this, we used electrocorticography intracranial recordings from epileptic patients performing an out loud or a silent reading task. In these tasks, short stories scrolled across a video screen in two conditions: subjects read the same stories both aloud (overt) and silently (covert). In a control condition the subject remained in a resting state. We first built a high gamma (70–150 Hz) neural decoding model to reconstruct spectrotemporal auditory features of self-generated overt speech. We then evaluated whether this same model could reconstruct auditory speech features in the covert speech condition. Two speech models were tested: a spectrogram and a modulation-based feature space. For the overt condition, reconstruction accuracy was evaluated as the correlation between original and predicted speech features, and was significant in each subject (*p* < 10^−5^; paired two-sample *t*-test). For the covert speech condition, dynamic time warping was first used to realign the covert speech reconstruction with the corresponding original speech from the overt condition. Reconstruction accuracy was then evaluated as the correlation between original and reconstructed speech features. Covert reconstruction accuracy was compared to the accuracy obtained from reconstructions in the baseline control condition. Reconstruction accuracy for the covert condition was significantly better than for the control condition (*p* < 0.005; paired two-sample *t*-test). The superior temporal gyrus, pre- and post-central gyrus provided the highest reconstruction information. The relationship between overt and covert speech reconstruction depended on anatomy. These results provide evidence that auditory representations of covert speech can be reconstructed from models that are built from an overt speech data set, supporting a partially shared neural substrate.

## Introduction

Mental imagery produces experiences and neural activation patterns similar to actual perception. For instance, thinking of moving a limb activates the motor cortex, internal object visualization activates the visual cortex, with similar effects observed for each sensory modality (Roth et al., 1996; Kosslyn et al., 2001; Kosslyn, 2005; Stevenson and Case, 2005). Auditory imagery is defined as the mental representation of sound perception in the absence of external auditory stimulation. Behavioral and neural studies have suggested that structural and temporal properties of auditory features, such as pitch (Halpern, 1989), timbre (Pitt and Crowder, 1992; Halpern et al., 2004), loudness (Intons-Peterson, 1980) and rhythm (Halpern, 1988) are preserved during music imagery (Hubbard, 2013). However, less is known about the neural substrate of speech imagery. Speech imagery (inner speech, silent speech, imagined speech, covert speech, or auditory verbal imagery) refers to our ability to “hear” speech internally without the intentional movement of any extremities, such as the lips, tongue, hands, or auditory stimulation (Brigham and Kumar, 2010).

The neural basis of speech processing has been a topic of intense investigation for over a century (Hickok and Poeppel, 2007). The functional cortical organization of speech comprehension includes Heschl's gyrus (primary auditory cortex), the superior temporal gyrus (STG), and sulcus (STS) (e.g., Wernicke's area). Speech production depends on premotor, motor and posterior inferior frontal regions (e.g., Broca's area) (Fiez and Petersen, 1998; Heim et al., 2002; Duffau et al., 2003; Billingsley-Marshall et al., 2007; Towle et al., 2008; Price, 2012). How these brain areas interact to encode higher-level components of speech such as phonological, semantic, or lexical features, as well as their role in covert speech, remains unclear. Increasing evidence suggests that speech imagery and perception activate the same cortical areas. Functional imaging studies (Yetkin et al., 1995; Rosen et al., 2000; Palmer et al., 2001). Transcranial magnetic stimulation over motor sites and inferior frontal gyrus induced speech arrest in both overt and covert speech production (Aziz-Zadeh et al., 2005). Finally, brain lesion studies have shown high correlation between overt and covert speech abilities, such as rhyme and homophones judgment (Geva et al., 2011b) for patients with aphasia.

Imagery-related brain activation could result from top-down induction mechanisms including memory retrieval (Kosslyn et al., 2001; Kosslyn, 2005) and motor simulation (Guenther et al., 2006; Price, 2011; Tian and Poeppel, 2012). In memory retrieval, perceptual experience may arise from stored information (objects, spatial properties, and dynamics) acquired during actual speech perception and production experiences (Kosslyn, 2005). In motor simulation, a copy of the motor cortex activity (efference copy) is forwarded to lower sensory cortices, enabling a comparison of actual with desired movement, and permitting online behavioral adjustments (Jeannerod, 2003; Tian and Poeppel, 2012). Despite findings of overlapping brain activation during overt and covert speech (Hinke et al., 1993; Yetkin et al., 1995; McGuire et al., 1996; Rosen et al., 2000; Palmer et al., 2001; Aleman, 2004; Aziz-Zadeh et al., 2005; Geva et al., 2011a), it is likely that covert speech is not simply overt speech without moving the articulatory apparatus. Behavioral judgment studies showed that aphasic patients indicated inner speech impairment, while maintaining relatively intact overt speech abilities, while others manifested the reverse pattern (Geva et al., 2011b). Similarly, imaging techniques showed different patterns of cortical activation during covert compared to overt speech, namely in the premotor cortex, left primary motor cortex, left insula, and left superior temporal gyrus (Huang et al., 2002; Shuster and Lemieux, 2005; Pei et al., 2011). This suggests that brain activation maps associated with both tasks are dissociated at least in some cases (Feinberg et al., 1986; Aleman, 2004; Shuster and Lemieux, 2005; Geva et al., 2011a,b,c). The extent to which auditory perception and imagery engage similar underlying neural representations remains poorly understood.

To investigate similarities between the neural representations of overt and covert speech, we employed neural decoding models to predict auditory features experienced during speech imagery. Decoding models predict information about stimuli or mental states from recorded neural activity (Bialek et al., 1991). This technique has attracted increasing interest in neuroscience as a quantitative method to test hypotheses about neural representation (Warland et al., 1997; Kay et al., 2008; Kay and Gallant, 2009; Naselaris et al., 2011; Pasley et al., 2012). For instance, decoding models have allowed predicting continuous limb trajectories (Carmena et al., 2003; Hochberg et al., 2006, 2012; Schalk et al., 2007; Pistohl et al., 2008) from the motor cortex. In the visual domain, visual scenes can be decoded from neural activity in the visual cortex (Warland et al., 1997; Kay et al., 2008). Similarly, this approach has been used to predict continuous spectrotemporal features of speech (Guenther et al., 2009; Mesgarani et al., 2009). We used this approach to compare decoding accuracy during overt and covert conditions in order to evaluate the similarity of speech representations during speech perception and imagery.

We hypothesized that speech perception and imagery share a partially overlapping neural representation in auditory cortical areas. We reasoned that if speech imagery and perception share neural substrates, the two conditions should engage similar neural representations. Thus, a neural decoding model trained from overt speech should be able to predict speech features in the covert condition. (Pasley et al., 2012) showed that auditory spectrotemporal features of speech could be accurately reconstructed, and used to identify individual words during various listening tasks. In this study, we used a similar neural decoding model trained on sounds from self-generated overt speech. This model was then used to decode spectrotemporal auditory features from brain activity measured during a covert speech condition. Our results provide evidence for a shared neural representation underlying speech perception and imagery.

To test these hypotheses we used electrocorticography (ECoG), which provides high spatiotemporal resolution recordings of non-primary auditory cortex (Leuthardt et al., 2004). In particular, the high gamma band (HG, ~70–150 Hz) reliably tracks neuronal activity in all sensory modalities (Lachaux et al., 2012) and correlates with the spike rate of the underlying neural population (Miller et al., 2007; Boonstra et al., 2009; Lachaux et al., 2012). HG activity in auditory and motor cortex has been linked to speech processing (Crone et al., 2001; Canolty, 2007; Towle et al., 2008; Llorens et al., 2011; Pasley et al., 2012), and served as the input signal for all tested neural decoding models.

## Materials and methods

### Subjects and data acquisition

**Figure 1:**
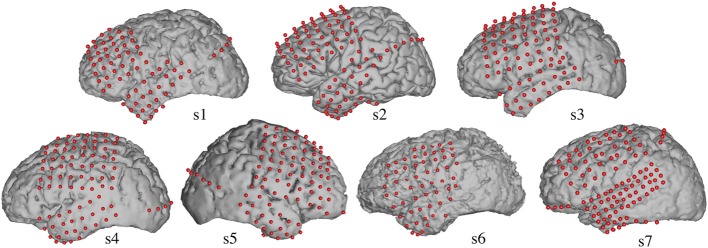
**Electrode locations**. Grid locations for each subject are overlaid on cortical surface reconstructions of each subject's MRI scan.

Electrocorticographic (ECoG) recordings were obtained using subdural electrode arrays implanted in 7 patients undergoing neurosurgical procedures for epilepsy (Table 1). All patients volunteered and gave their informed consent (approved by the Albany Medical College Institutional Review Board) before testing. The implanted electrode grids (Ad-Tech Medical Corp., Racine, WI; PMT Corporation, Chanhassen, MN) consisted of platinum–iridium electrodes (4 mm in diameter, 2.3 mm exposed) that were embedded in silicon and spaced at an inter-electrode distance of 0.6–1 cm. Grid placement and duration of ECoG monitoring were based solely on the requirements of the clinical evaluation (Figure 1).

**Table 1:** **Clinical profiles of subjects**.

**Subject**	**Age**	**Sex**	**Handed-ness**	**FSIQ**	**VIQ**	**PIQ**	**LL**	**Seizure focus**	**Grid/Strip locations and contact numbers**
S1	30	M	Right	74	64	90	Bi-lateral	Left temporal	Left temporal (35)
									Left temporal pole (4)
									Left fronto-parietal (48)
									Left occipital pole (4)
S2	29	F	Right	90	91	90	Left	Left temporal	Left temporal (35)
									Left fronto-parietal (56)
									Left temporal (4)
									Left occipital pole (4)
S3	26	F	Right	112	106	117	Left	Left temporal	Left temporal (35)
									Left fronto-parietal (64)
									Left temporal (4)
									Left occipital pole (4)
S4	56	M	Right	84	82	87	Left	Left temporal	Left temporal (35)
									Left fronto-parietal (56)
									Left occipital pole (4)
S5	26	M	Right	102	103	100	Left	Right temporal	Right temporal (35)
									Right fronto-parietal (64)
									Right frontal pole (6)
									Right occipital pole (6)
S6	45	M	Right	98	93	105	Left	Left frontal	Left front-temporal (54)
									Left temporal (4)
S7	29	F	Right	84	111	95	Bi-lateral	Left temporal	Left temporal (68)
									Left fronto-parietal (40)
									Left frontal pole (4)
									Left parietal (4)
									Left temporal (4)

*All of the subjects had normal cognitive capacity and were functionally independent. Full scale (FSIQ), verbal (VIQ), and performance (PIQ) intelligence has was based on the Wechsler Adult Intelligence Scale (WAIS-III) test. Language lateralization (LL) was based on the Wada test*.

ECoG signals were recorded at the bedside using seven 16-channel g.USBamp biosignal acquisition devices (g.tec, Graz, Austria) at a sampling rate of 9600 Hz. Electrode contacts distant from epileptic foci and areas of interest were used for reference and ground. Data acquisition and synchronization with the task presentation were accomplished using BCI2000 software (Schalk et al., 2004; Schalk, 2010). All channels were subsequently downsampled to 1000 Hz, corrected for DC shifts, and band pass filtered from 0.5 to 200 Hz. Notch filters at 60, 120, and 180 Hz were used to remove electromagnetic noise. The time series were then visually inspected to remove the intervals containing ictal activity as well as channels that had excessive noise (including broadband electromagnetic noise from hospital equipment or poor contact with the cortical surface). Finally, electrodes were re-referenced to a common average. The high gamma frequency band (70–150 Hz) was extracted using the Hilbert transform.

In addition to the ECoG signals, we acquired the subject's voice through a dynamic microphone (Samson R21s) that was rated for voice recordings (bandwidth 80–12000 Hz, sensitivity 2.24 mV/Pa) and placed within 10 cm of the patient's face. We used a dedicated 16-channel g.USBamp to amplify and digitize the microphone signal in sync with the ECoG data. Finally, we verified the patient's compliance in the covert task using an eye-tracker (Tobii T60, Tobii Sweden).

### Experimental paradigms

The recording session included three conditions. In the first condition, text excerpts from historical political speeches or a children's story [i.e., Gettysburg Address (Roy and Basler, 1955), JFK's Inaugural Address (Kennedy, 1961), or Humpty Dumpy (Mother Goose's Nursery Rhymes, 1867)] were visually displayed on the screen moving from right to left at the vertical center of the screen. The rate of scrolling text ranged between 42 and 76 words/min, and was adjusted based on the subject's attentiveness, cognitive/verbal ability, and comfort prior to experimental recordings. In the first condition, the subject was instructed to read the text aloud (overt condition). In the second condition, the same text was displayed at the same scrolling rate, but the subject was instructed to read it silently (covert condition). The third condition served as the control and was obtained while the subject was in a resting state condition (baseline control). For each condition, a run lasted between 6 and 8 min, and was repeated 2–3 times depending on the mental and physical condition of the subjects.

### Auditory speech representations

We evaluated the predictive power of a neural decoding model based on high gamma signals (see section Decoding Model and Reconstruction Procedure for details) to reconstruct two auditory feature representations: a spectrogram-based and a modulation-based representation. The spectrogram is a time-varying representation of the amplitude envelope at each acoustic frequency. This representation was generated by an affine wavelet transform of the sound pressure waveform using a 128 channel-auditory filter bank mimicking the frequency analysis of the auditory periphery (Chi et al., 2005). The 128 acoustic frequencies of the initial spectrograms were subsequently downsampled to 32 acoustic frequency bins—with logarithmically spaced center frequencies ranging from 180 to 7000 Hz.

The modulation representation is based on a non-linear transformation of the spectrogram. Spectral and temporal fluctuations reflect important properties of speech intelligibility. For instance, comprehension is impaired when temporal modulations (<12 Hz) or spectral modulations (4 cycles/kHz) are removed (Elliott and Theunissen, 2009). In addition, low and intermediate temporal modulation rates (<4 Hz) are linked with syllable rate, whereas fast modulations (>16 Hz) are related to syllable onsets and offsets. Similarly, broad spectral modulations are associated with vowel formants, whereas narrow spectral modulations are associated with harmonics (Shamma, 2003). The modulation representation was generated by a 2-D affine wavelet transform of the 128 channel auditory spectrogram. The bank of modulation-selective filters spanned a range of spectral scales (0.5–8 cycle/octave) and temporal rates (1–32 Hz), and was estimated from studies of the primary auditory cortex (Chi et al., 1999). The modulation representation was obtained by taking the magnitude of the complex-valued output of the filter bank, and subsequently reduced to 60 modulation features (5 scales × 12 rates) by averaging along the frequency dimension. These operations were computed using the NSL Matlab toolbox (http://www.isr.umd.edu/Labs/NSL/Software.htm). In summary, the neural decoding model predicted 32 spectral frequency features and 60 rate and scale features in the spectrogram-based and modulation-based speech representation, respectively.

### Decoding model and reconstruction procedure

#### Overt speech decoding

**Figure 2:**
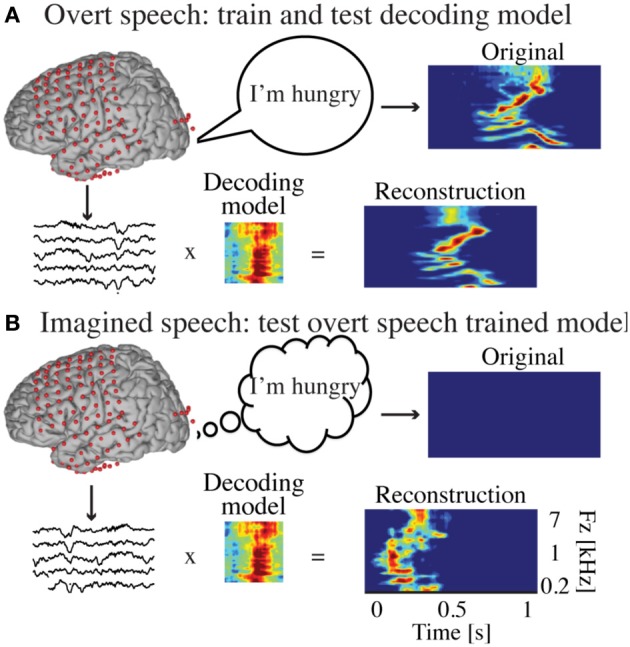
**Decoding approach. (A)** The overt speech condition was used to train and test the accuracy of a neural-based decoding model to reconstruct spectrotemporal features of speech. The reconstructed patterns were compared to the true original (spoken out loud) speech representation (spectrogram or modulation-based). **(B)** During covert speech, there is no behavioral output, which prevents building a decoding model directly from covert speech data. Instead, the decoding model trained from the overt speech condition is used to decode covert speech neural activity. The covert speech reconstructed patterns were compared to identical speech segments spoken aloud during the overt speech condition (using dynamic time warping realignment).

The decoding model was a linear mapping between neural activity and the speech representation (Figure 2A). It modeled the speech representation (spectrogram or modulation) as a linear weighted sum of activity at each electrode as follows:

(1) $$\hat{S}(\text{t},\text{p})=\sum_{\tau }\sum_{\text{n}}\text{g}(\tau ,\text{p},\text{n})\text{R}(\text{t}-\tau ,\text{n}),$$

where R(t − τ, n) is the high gamma activity of electrode n at time (t − τ), where τ is the time lag ranging between −500 and 500 ms. $\hat{\text{S}}$(t, p) is the estimated speech representation at time t and speech feature p, where p is one of 32 acoustic frequency features in the spectrogram-based representation (Figure 5B) and one of 60 modulation features (5 scales × 12 rates) in the modulation-based representation (Figure 7B; see section Auditory Speech Representations for details). Finally, g(τ, p, n) is the linear transformation matrix, which depends on the time lag, speech feature, and electrode channel. Both speech representations and the neural high gamma response data were synchronized, downsampled to 100 Hz, and standardized to zero mean and unit standard deviation prior to model fitting.

Model parameters, the matrix g described above, were fit using gradient descent with early stopping regularization—an iterative linear regression algorithm. We used a jackknife resampling technique to fit separately between 4 and 7 models (Efron, 1982), and then averaged the parameter estimates to yield the final model. To maintain the temporal correlations within neural activity and speech features, the data were first divided into 7 seconds blocks. From these blocks, 90% were randomly partitioned into a training set and 10% into a testing set. Within the training set, 10% of the data were used to monitor out-of-sample prediction accuracy to determine the early stopping criterion and minimize overfitting. The algorithm was terminated after a series of 30 iterations failing to improve performance. Finally, model prediction accuracy (see section Evaluation for details) was evaluated on the independent testing set. Model fitting was performed using the STRFLab MATLAB toolbox (http://strflab.berkeley.edu/).

#### Covert speech decoding

Decoding covert speech is complicated by the lack of any measurable behavioral or acoustic output that is synchronized to brain activity. In other words, there is no simple ground truth by which to evaluate the accuracy of the model when a well-defined output is unavailable. To address this, we used the following approach. First, the decoding model was trained using data from the overt speaking condition. Second, the same model (Equation 1) was applied to data from the covert condition to predict speech features imagined by the subject (Figure 2B), as follows:

(2) $${\hat{S}}_{covert}(\text{t},\text{p})=\sum_{\tau }\sum_{\text{n}}\text{g}(\tau ,\text{p},\text{n}){\text{R}}_{covert}(\text{t}-\tau ,\text{n}),$$

where *Ŝ*_*covert*_(t, p) is the predicted covert speech representation at time t and speech feature p, and R_*covert*_(t−τ, n) is the high gamma neuronal response of electrode n at time (t−τ), where τ is the time lag ranging between −500 and 500 ms. Finally, g(τ, p, n) is the linear model trained from the overt speech condition. To evaluate prediction accuracy during covert speech, we made the assumption that the covert speech representation should match the spectrotemporal content of overt speech. In this sense, overt speech is used as the “ground truth.” Because subjects read the same text segments in both overt and covert conditions, we computed the similarity between the covert reconstructions and the corresponding original speech sounds recorded during the overt condition. To account for timing differences between conditions, we used dynamic time warping to realign the covert reconstruction to the original overt speech sound, as described in the next section.

#### Dynamic time warping

**Figure 3:**
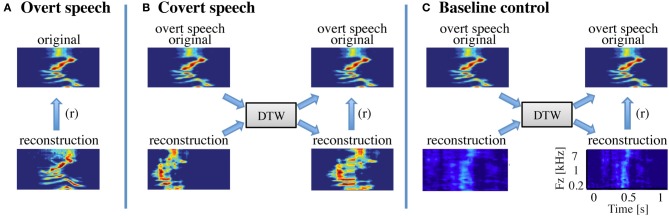
**Speech realignment. (A)** Overt speech analysis—the overall reconstruction accuracy for the overt speech condition was quantified by computing directly the correlation coefficient (Pearson's r) between the reconstructed and original speech representations **(B)** Covert speech analysis—the covert speech reconstruction is not necessarily aligned to the corresponding overt speech representation due to speaking rate differences and repetition irregularities. The reconstruction was thus realigned to the overt speech stimuli using dynamic time warping. The overall reconstruction accuracy was then quantified by computing the correlation coefficient (Pearson's r) between the covert speech reconstruction and the original speech representation. **(C)** Baseline control analysis—a resting state (baseline control) condition was used to assess statistical significance of covert speech reconstruction accuracy. Resting state activity was used to generate a noise reconstruction and dynamic time warping was applied to align the noise reconstruction to overt speech as in **(B)**. Because dynamic time warping has substantial degrees of freedom, due to its ability to stretch and compress speech segments, the overall reconstruction accuracy for the baseline control condition is significantly higher than zero. However, direct statistical comparisons between the covert and baseline conditions are valid as equivalent analysis procedures are applied to both covert and resting state neural data.

We used a dynamic time warping (DTW) algorithm to realign the covert speech reconstruction with the corresponding spoken audio signal from the overt condition, allowing a direct estimate of the covert reconstruction accuracy (Figure 3B). For the overt speech reconstructions, dynamic time warping was not employed (Figure 3A), unless otherwise stated. DTW is a standard algorithm used to align two sequences that may vary in time or speed (Sakoe and Chiba, 1978; Giorgino, 2009). The idea behind DTW is to find the optimal path through a local similarity matrix d, computed between every pair of elements in the query and template time series, X ∈ ℝ^P x N^ and Y ∈ ℝ^P x M^ as follows:

(3) $$\text{d}\left(\text{n},\text{ m}\right)=\text{f}\left({\text{x}}_{\text{n}},{\text{y}}_{\text{m}}\right),\text{ d}\in {\mathbb{R}}^{\text{N x M}},$$

where d is the dissimilarity matrix at time n and m, f can be any distance metric between sequence x and y at time n and m, respectively. In this study, we used the Euclidean distance, defined as $\text{d}\left(\text{n},\text{m}\right)=\sqrt{\sum_{p}^{P}{\left(x_{np}-y_{mp}\right)}^{2}}$. Given φ, the average accumulated distortion between both warped signals is defined by:

(4) $${\text{d}}_{\varphi }(\text{x},\text{ y})=\sum_{\text{k = 1}}^{\text{K}}\frac{\text{d}\left(\varphi_{\text{x}}(\text{k}),\varphi_{\text{y}}(\text{k})\right)}{{\text{C}}_{\varphi }},$$

where φ_x_ and φ_y_ are the warping functions of length K (that remap the time indices of X and Y, respectively), and C_φ_ is the corresponding normalization constant (in this case N + M), ensuring that the accumulated distortions are comparable along different paths. The optimal warping path φ, chooses the indices of X and Y in order to minimize the overall accumulated distance.

(5) $$\text{D}\left(\text{X},\text{ Y}\right)=\underset{{}_{\varphi }}{\min}{\text{d}}_{\varphi }\left(\text{X},\text{ Y}\right),$$

where D is the accumulated distance or global dissimilarity. The alignment was computed using Rabiner-Juan step patterns (type 3) (Rabiner, 1993). This step pattern constrained the sets of allowed transitions between matched pairs to:

(6) $$\left[\varphi_{x}\left(\text{k}+1\right)-\varphi_{\text{x}}\left(\text{k}\right),\varphi_{\text{y}}\left(\text{k}+1\right)-\varphi_{\text{y}}\left(\text{k}\right)]\in \{(1,2),(2,1),(1,1)\right\}$$

In addition, we assumed that the temporal offsets between covert speech and original overt speech would be less than 2 s, and thus introduced a global constraint—the Sakoe-Chiba band window (Sakoe and Chiba, 1978), defined as follows:

(7) $$\left|\varphi_{\text{x}}\left(\text{k}\right)-\varphi_{\text{y}}(\text{k})\right|\leq \text{T}$$

where *T* = 2 s was the chosen value that defines the maximum-allowable width of the window. Finally, to reduce computational load, the entire time series was broken into 30 s segments, and warping was applied on each individual pair of segments (overt, covert, or baseline control reconstruction warped to original speech representation). The warped segments were concatenated and the reconstruction accuracy was defined on the full time series of warped data. The DTW package in R (Giorgino, 2009) was used for all analyses.

#### Baseline control condition (resting state)

To assess statistical significance of the covert reconstruction accuracy, we applied the same decoding steps (sections Covert speech decoding—Dynamic time warping) to a baseline control condition taken from data recorded during a separate resting state recording session. The overt speech decoding model was applied to neural data from the baseline control, as follows:

(8) $${\hat{S}}_{baseline}(\text{t},\text{p})=\sum_{\tau }\sum_{\text{n}}\text{g}(\tau ,\text{p},\text{n}){\text{R}}_{baseline}(\text{t}-\tau ,\text{n}),$$

where **Ŝ**_*baseline*_(t, p) is the predicted baseline reconstruction at time t and speech feature p, and R_*baseline*_(t−τ, n) is the high gamma neural response during resting state. Finally, g(τ, p, n) is the linear model trained from the overt speech condition. We also used DTW to realign the baseline control reconstruction with the spoken audio signal from the overt condition, allowing a direct estimate of the control condition decoding predictions (Figure 3C).

### Evaluation

In the overt speech condition, reconstruction accuracy was quantified by computing the correlation coefficient (Pearson's r) between the reconstructed and original speech representation using data from the independent test set. For each cross-validation resample, we calculated one correlation coefficient for each speech feature over time—leading to 32 correlation coefficients (one for each acoustic frequency features) for the spectrogram-based model and 60 correlation coefficients (5 scale × 12 rate features) for the modulation-based model. Overall reconstruction accuracy was reported as the mean correlation over resamples and speech components (32 and 60 for the spectrogram and modulation representation, respectively). Standard error of the mean (s.e.m.) was calculated by taking the standard deviation of the overall reconstruction accuracy across resamples. To assess statistical significance (see section Statistics for details), overt speech reconstruction accuracy was compared to the accuracy obtained from the baseline control condition (resting state).

In the covert speech condition, we first realigned the reconstructions and original overt speech representations using dynamic time warping (Figure 3B). Then, we computed the overall reconstruction accuracy using the same procedure as in the overt speech condition. To evaluate statistical significance (see section Statistics for details), DTW was also applied to the baseline control condition prior to assessing the overall reconstruction accuracy (Figure 3C).

To further assess the predictive power of the reconstruction process, we evaluated the ability to identify specific blocks of speech utterances within the continuous recording (Figure 11). First, 24–140 segments of speech utterances (5 s duration) were extracted from the original and reconstructed spectrogram representations. Second, a confusion matrix was constructed where each element contained the similarity score between the target reconstructed segment and the original reference segments from the overt speech spectrogram. To compute the similarity score between each target and reference segment, DTW was applied to temporally align each pair and the mean correlation coefficient was used as the similarity score. The confusion matrix reflects how well a given reconstructed segment matches its corresponding original segment vs. other candidates. The similarity scores were sorted, and identification accuracy was quantified as the percentile smaller than the rank of the correct segment (Pasley et al., 2012). At chance level, the expected percentile rank is 0.5, while perfect identification is 1.0.

To define the most informative areas for overt speech decoding accuracy, we isolated for each electrode its corresponding decoding weights, and used the electrode-specific weights to generate a separate reconstruction for each electrode. This allowed calculating a reconstruction accuracy correlation coefficient for each individual electrode. We applied the same procedure to the baseline condition. Baseline reconstruction accuracy was subtracted from the overt values to generate subject-specific informative area maps (Figure 8). The same technique was used in the covert speech condition, except that DTW was applied to realign separately each electrode-specific reconstruction to the original overt speech. Similarly, baseline reconstruction accuracy (with DTW realignment) was subtracted from the covert values to define the informative areas (Figure 12).

### Statistics

To assess statistical significance for the difference between overt speech and baseline control reconstruction accuracy, we used Hotelling's *t* statistic with a significance level of *p* < 10^−5^. This test accounts for the dependence of the two correlations on the same group (i.e., both correlations are relative to the same original overt speech representation) (Hotelling, 1940; Birk, 2013). It evaluates whether the correlations between overt speech reconstruction accuracy and baseline reconstruction accuracy differed in magnitude taking into account their intercorrelation, as follows:

(9) $$t=\frac{\left(r_{jk}-r_{jh}\right)\sqrt{\left(n-3)(1+r_{kh}\right)}}{\sqrt{2|R|}}$$

where *r*_*jk*_ is the correlation between original overt speech and reconstruction, *r*_*jh*_ is the correlation between original overt speech and baseline reconstruction and *r*_*kh*_ is the correlation between overt speech reconstruction and baseline reconstruction; *df* = *n* − 3 is the effective sample size (Kaneoke et al., 2012) and where

(10) $$|R|=1+2r_{jk}\;r_{jh}\;r_{kh}-r_{jk}^{2}-r_{jh}^{2}-r_{kh}^{2}$$

At the population level (Figure 5A), statistical significance was performed using Student's *t*-tests (*p* < 10^−5^) after first applying Fisher's Z transform to convert the correlation coefficients to a normal distribution (Fisher, 1915).

Test of significance in the covert speech condition was equivalent to the overt condition (Equation 9; *p* < 0.05; Hotelling's *t*-test), except that the reconstructions and original overt speech representations were first realigned using dynamic time warping. Since DTW induces an artificial increase in correlation by finding an optimal warping path between any two signals (including potential noise signals), this procedure causes the accuracy for baseline reconstruction to exceed zero correlation. However, because the equivalent data processing sequence was applied to both conditions, any statistical differences between the two conditions were due to differences in the neural input signals.

At the population level (Figure 9), we directly compared the reconstruction accuracy in all three conditions (overt, covert and baseline control). DTW realignment to the original overt speech was first applied separately for each condition. Reconstruction accuracy was computed as the correlation between the respective realigned pairs. Statistical significance was performed using Fisher's Z transform and One-Way ANOVA (*p* < 10^−6^), followed by *post-hoc t*-test (*p* < 10^−5^ for overt speech; *p* < 0.005 for covert speech).

For individual subjects, significance of identification rank was computed using a randomization test (*p* < 10^−5^ for overt speech; *p* < 0.005 for covert speech; *p* > 0.5 for baseline control). We shuffled the segment label in the candidate set 10,000 times to generate a null distribution of identification ranks under the hypothesis that there is no relationship between target and reference speech segments. Time-varying speech representations are auto-correlated. To maintain temporal correlations in the data, and preserve the exchangeability of the trial labels, the length of the extracted segments was chosen sufficiently longer than the speech representation autocorrelation (5 s). The proportion of shuffled ranks greater than the observed rank yields the *p*-value that the observed accuracy is due to chance. Identification accuracy was assessed for each of the three experimental conditions (overt reconstruction, covert reconstruction, baseline control reconstruction). At the population level, significant identification performance was tested using a one-sided, one-sample *t*-test (*p* < 10^−5^ for overt speech; *p* < 0.05 for covert speech; *p* > 0.5 for baseline control).

For the informative electrode analysis, statistical significance of overt speech reconstruction was determined relative to the baseline condition using Hotelling's t statistic (Equation 9; Hotelling's *t*-test). Electrodes were defined as “informative” if the overt speech reconstruction accuracy was significantly greater than baseline (*p* < 0.05; Hotelling's *t*-test with Bonferroni correction). The same procedure was used for covert speech informative areas (Equation 9; *p* < 0.05; Hotelling's *t*-test with Bonferroni correction), except that DTW was used in both covert speech and baseline control condition.

To investigate possible anatomical differences between overt and covert informative areas, all significant electrodes (either overt, covert or both conditions; *p* < 0.05; Bonferroni correction) were selected for an unbalanced Two-Way ANOVA, with experimental condition (overt and covert) and anatomical region (superior temporal gyrus, pre- and post-central gyrus) as factors. Figure 13 shows significant electrodes in these regions across subjects, co-registered with the Talairach brain template (Lancaster et al., 2000).

### Coregistration

**Figure 4:**
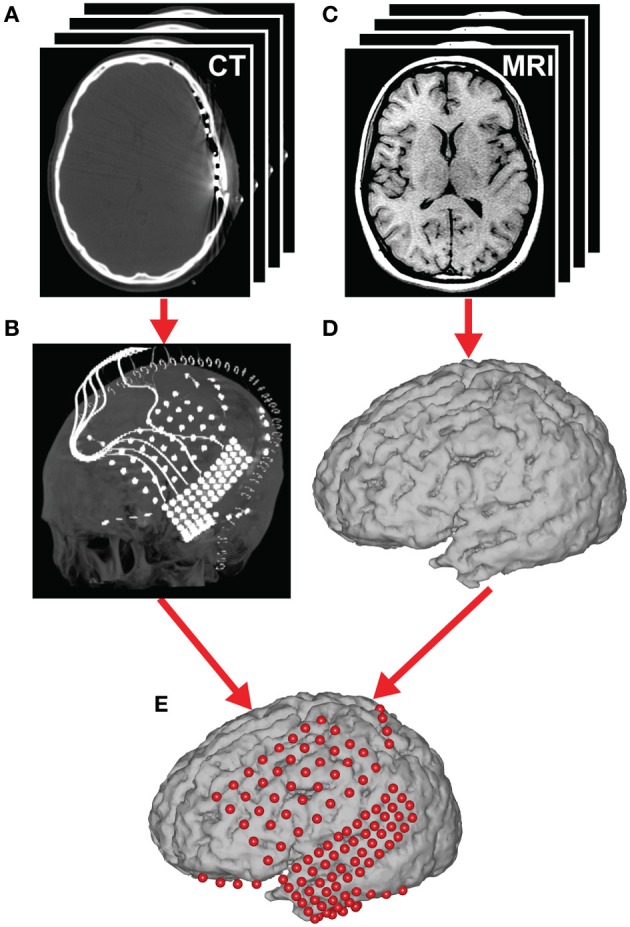
**Brain mapping and electrode localization. (A)** Post-operative CT scans (1 mm slices) and **(C)** pre-operative structural MRI scans (1.5 mm slices, T1-weighted) were acquired for each subject. From these scans, grid position **(B)** and the cortical surface **(D)** were reconstructed providing a subject-specific anatomical model **(E)** (see section Coregistration for details).

Each subject had post-operative anterior–posterior and lateral radiographs (Figure 4), as well as computer tomography (CT) scans to verify ECoG grid locations. Three-dimensional cortical models of individual subjects were generated using pre-operative structural magnetic resonance (MR) imaging. These MR images were co-registered with the post-operative CT images using Curry software (Compumedics, Charlotte, NC) to identify electrode locations. Electrode locations were assigned to Brodmann areas using the Talairach Daemon (http://www.talairach.org, (Lancaster et al., 2000). Activation maps computed across subjects were projected on this 3D brain model, and were generated using a custom Matlab program (Gunduz et al., 2012).

## Results

### Overt speech

#### Spectrogram-based reconstruction

**Figure 5:**
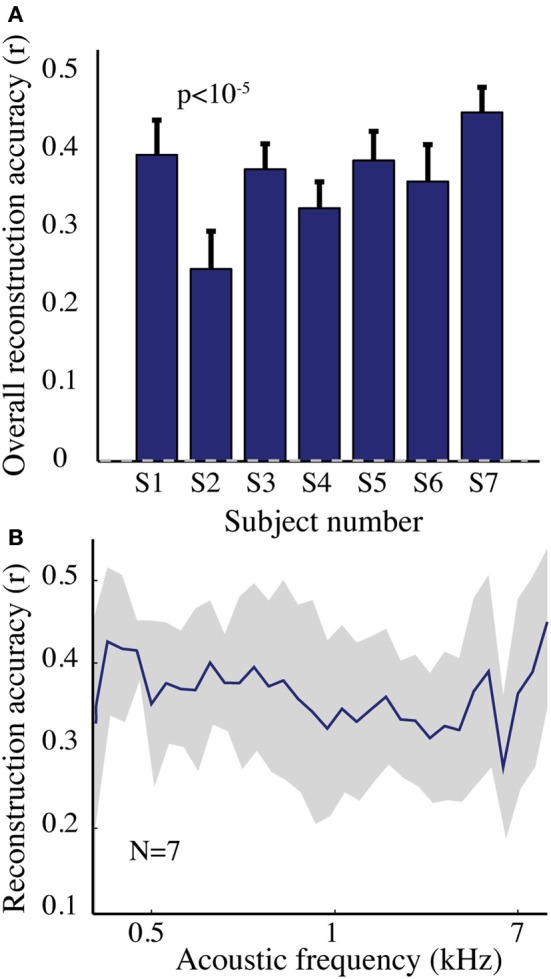
**Overt speech reconstruction accuracy for the spectrogram-based speech representation. (A)** Overall reconstruction accuracy for each subject using the spectrogram-based speech representation. Error bars denote standard error of the mean (s.e.m.). Overall accuracy is reported as the mean over all features (32 acoustic frequencies ranging from 0.2–7 kHz). The overall spectrogram reconstruction accuracy for the overt speech was greater than baseline control reconstruction accuracy in all individuals (*p* < 10^−5^; Hotelling's *t*-test). Baseline control reconstruction accuracy was not significantly different from zero (*p* > 0.1; one-sample *t*-test; gray dashed line) **(B)** Reconstruction accuracy as a function of acoustic frequency averaged over all subjects (*N* = 7) using the spectrogram model. Shaded region denotes s.e.m. over subjects.

**Figure 6:**
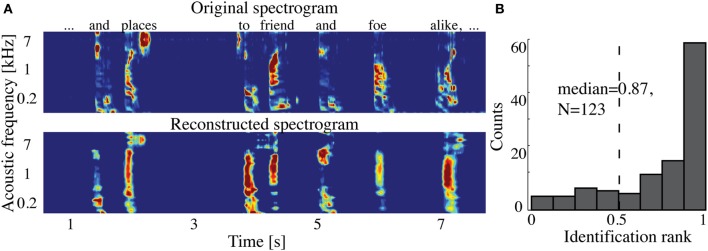
**Overt speech reconstruction and identification. (A)** Top panel: segment of the original sound spectrogram (subject's own voice), as well as the corresponding text above it. Bottom panel: same segment reconstructed with the decoding model. **(B)** Identification rank. Speech segments (5 s) were extracted from the continuous spectrogram. For each extracted segment (*N* = 123) a similarity score (correlation coefficient) was computed between the target reconstruction and each original spectrogram of the candidate set. The similarity scores were sorted and identification rank was quantified as the percentile rank of the correct segment. 1.0 indicates the target reconstruction matched the correct segment out of all candidate segments; 0.0 indicates the target was least similar to the correct segment among all other candidates; (dashed line indicates chance level = 0.5; median identification rank = 0.87; *p* < 10^−5^; randomization test).

The overall spectrogram reconstruction accuracy for overt speech was significantly greater than baseline control reconstruction accuracy in all individual subjects (*p* < 10^−5^; Hotelling's *t*-test, Figure 5A). At the population level, mean overall reconstruction accuracy averaged across all subjects (*N* = 7) was also significantly higher than baseline control condition (*r* = 0.41, *p* < 10^−5^; Fisher's Z transform followed by paired two-sample *t*-test). The baseline control reconstruction accuracy was not significantly different from zero (*r* = 0.0, *p* > 0.1; one-sample *t*-test; dashed line; Figure 5A). Group averaged reconstruction accuracy for individual acoustic frequencies ranged between *r* = ~0.25–0.5 (Figure 5B). An example of a continuous segment of the original and reconstructed spectrogram is depicted for a subject with left hemispheric coverage in Figure 6A. In this subject, the reconstruction quality permitted accurate identification of individual decoded speech segments (Figure 6B). The median identification rank (0.87, *N* = 123 segments) was significantly greater than chance level (0.5, *p* < 10^−5^; randomization test). Identification performance was significant in each individual subject (*p* < 10^−5^; randomization test). Across all subjects, identification performance was significant for overt speech reconstruction (Figure 11; rank_*overt*_ = 0.91 > 0.5, *p* < 10^−6^; one-sided one-sample *t*-test), whereas the baseline control condition was not significantly greater than chance level (rank_*baseline*_ = 0.48 > 0.5, *p* > 0.5 one-sided one-sample *t*-test).

#### Modulation-based reconstruction

**Figure 7:**
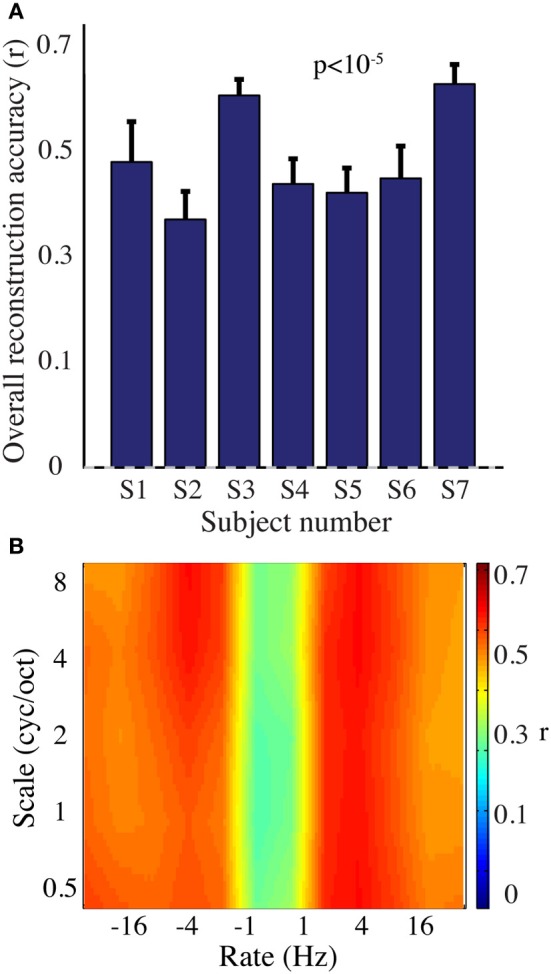
**Overt speech reconstruction accuracy for the modulation-based speech representation. (A)** Overall reconstruction accuracy for each subject using the modulation-based speech representation. Error bars denote s.e.m. Overall accuracy is reported as the mean over all features (5 spectral and 12 temporal modulations ranging between 0.5–8 cyc/oct and -32-32 Hz, respectively). The overall modulation reconstruction accuracy for the overt speech was greater than baseline control reconstruction accuracy in all individuals (*p* < 10^−5^; Hotelling's *t*-test). Baseline control reconstruction accuracy was not significantly different from zero (*p* > 0.1; one-sample *t*-test; gray dashed line). **(B)** Reconstruction accuracy as a function of rate and scale averaged over all subjects (*N* = 7).

We next evaluated reconstruction accuracy of the modulation representation. The overall reconstruction accuracy was significant in all individual subjects (*p* < 10^−5^; Hotelling's *t*-test Figure 7A). At a population level, mean overall reconstruction accuracy averaged over all patients (*N* = 7) was also significantly higher than the baseline reconstruction (*r* = 0.55, *p* < 10^−5^; Fisher's Z transform followed by paired two-sample *t*-test). The baseline control reconstruction accuracy was not significantly different from zero (*r* = 0.02, *p* > 0.1; one-sample *t*-test; dashed line; Figure 7A). Group averaged reconstruction accuracy for individual rate and scale was highest for temporal modulations above 2 Hz (Figure 7B).

#### Informative areas

**Figure 8:**
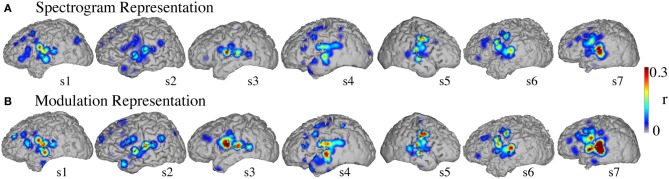
**Overt speech informative areas**. Reconstruction accuracy correlation coefficients were computed separately for each individual electrode and for both overt and baseline control conditions (see section Overt Speech: Informative areas for details). The plotted correlation values are calculated by subtracting the correlation during baseline control from the overt condition. The informative area map was thresholded to *p* < 0.05 (Bonferroni correction) **(A)** Spectrogram-based reconstruction accuracy **(B)** modulation-based reconstruction accuracy.

Figure 8 shows the significant informative areas (map thresholded at *p* < 0.05; Bonferroni correction), quantified by the electrode-specific reconstruction accuracy (see section Evaluation for details). In both spectrogram and modulation-based representations the most accurate sites for overt speech decoding were localized to the superior temporal gyrus, pre and post-central gyrus, consistent with previous spectrogram decoding studies (Pasley et al., 2012).

### Covert speech

#### Spectrogram-based reconstruction

**Figure 9:**
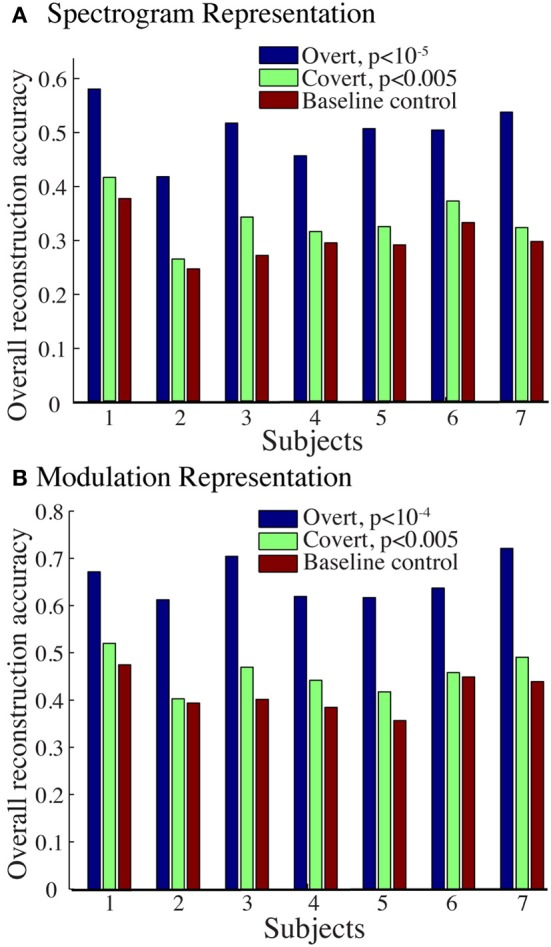
**Overall reconstruction accuracy using dynamic time warping realignment**. Overall reconstruction accuracy for each subject during overt speech, covert speech, and baseline control conditions after dynamic time warping realignment. **(A)** Spectrogram-based representation **(B)** Modulation-based representation.

**Figure 10:**
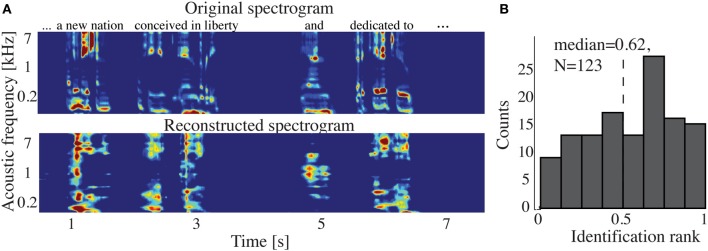
**Covert speech reconstruction. (A)** Top panel: a segment of the overt (spoken out loud) spectrogram representation. Bottom panel: the same segment reconstructed from neural activity during the covert condition using the decoding model. **(B)** Identification rank. Speech segments (5 s) were extracted from the continuous spectrogram. For each target segment (*N* = 123) a similarity score (correlation coefficient) was computed between the target reconstruction and each original spectrogram in the candidate set. The similarity scores were sorted and identification rank was quantified as the percentile rank of the correct segment. 1.0 indicates the target reconstruction matched the correct segment out of all candidate segments; 0.0 indicates the target was least similar to the correct segment among all other candidates. (dashed line indicates chance level = 0.5; median identification rank = 0.62; *p* < 0.005; randomization test).

**Figure 11:**
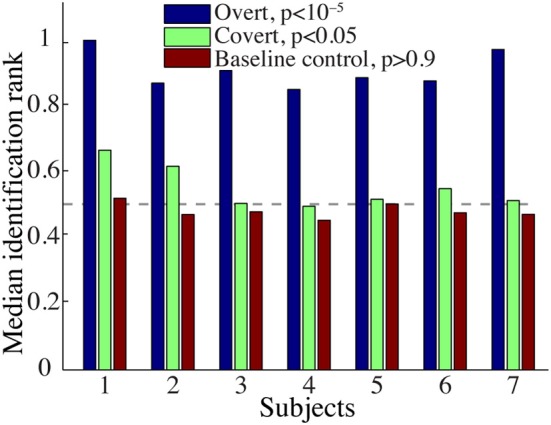
**Overt and covert speech identification**. Median identification rank for each subject during overt speech, covert speech, and baseline control conditions (see section Evaluation for more details). At the group level, rank_*overt*_ = 0.91 and rank_*covert*_ = 0.55 are significantly higher than chance level (0.5; randomization; gray dashed line), whereas rank_*baseline*_ = 0.48 is not significantly different.

Figure 9A shows the overall reconstruction accuracy for overt speech, covert speech, and baseline control after DTW realignment to the original overt speech was applied separately for each condition. The overall reconstruction accuracy for covert speech was significantly higher than the control condition in 5 out of 7 individual subjects (*p* < 0.05; Hotelling's *t*-test; *p* > 0.05 for the non-significant subjects). At the population level, there was a significant difference in the overall reconstruction accuracy across the three conditions [overt, covert and baseline control; *F*_(2, 18)_ = 35.3, *p* < 10^−6^; Fisher's Z transform followed by One-Way ANOVA]. *Post-hoc t*-tests confirmed that covert speech reconstruction accuracy was significantly lower than overt speech reconstruction accuracy (*r*_*covert*_ = 0.34 < *r*_*overt*_ = 0.50, *p* < 10^−5^; Fisher's Z transform followed by paired two-sample *t*-test), but higher than the baseline control condition (*r*_*covert*_ = 0.34 > *r*_*baseline*_ = 0.30, *p* < 0.005; Fisher's Z transform followed by a paired two-sample *t*-test). Figure 10A illustrates a segment of the reconstructed covert speech spectrogram and its corresponding overt segment (realigned with DTW). We next evaluated identification performance (*N* = 123 segments) for covert speech and baseline control conditions in this subject (Figure 10B). In the covert speech condition, the median identification rank equaled 0.62, and was significantly higher than chance level of 0.5 (*p* < 0.005; randomization test), whereas the baseline control condition was not significant (median identification rank = 0.47, *p* > 0.5; randomization test). Several of the remaining subjects exhibited a trend toward higher identification performance, but were not significant at the *p* < 0.05 level (Figure 11; randomization test). At the population level, mean identification performance across all subjects was significantly greater than chance for the covert condition (rank_*covert*_ = 0.55 > 0.5, *p* < 0.05; one-sided one-sample *t*-test), and not significant for the baseline control (rank_*baseline*_ = 0.48 > 0.5, *p* > 0.5; one-sided one-sample *t*-test). These results provide preliminary evidence that neural activity during auditory speech imagery can be used to decode spectrotemporal features of covert speech.

#### Modulation-based reconstruction

Reconstruction accuracy for the modulation-based covert speech condition was significant in 4 out of 7 individuals (*p* < 0.05; Hotelling's *t*-test; *p* > 0.1 for non-significant subjects; Figure 9B). At the population level, the overall reconstruction accuracy across the three conditions (overt, covert, and baseline control) was significantly different [*F*_(2, 18)_ = 62.1, *p* < 10^−6^; One-Way ANOVA]. *Post-hoc t*-tests confirmed that covert speech reconstruction accuracy was significantly lower than overt speech reconstruction accuracy (*r*_*covert*_ = 0.46 < *r*_*overt*_ = 0.66, *p* < 10^−5^; Fisher's Z transform followed by a paired two-sample *t*-test), but higher than the baseline control condition (*r*_*covert*_ = 0.46 > *r*_*baseline*_ = 0.42, *p* < 0.005; Fisher's Z transform followed by a paired two-sample *t*-test).

#### Informative areas

**Figure 12:**
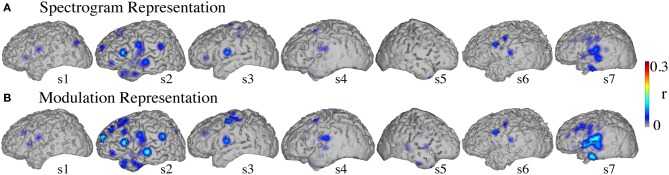
**Covert speech informative areas**. Reconstruction accuracy correlation coefficients were computed separately for each individual electrode and for both covert and baseline control conditions (see section Overt Speech: Informative areas and Covert Speech: Informative areas for details). The plotted correlation values are calculated by subtracting the correlation during baseline control from the covert condition. The informative area map was thresholded to *p* < 0.05 (Bonferroni correction) **(A)** Spectrogram-based reconstruction accuracy **(B)** modulation-based reconstruction accuracy.

Significant informative areas (map thresholded at *p* < 0.05; Bonferroni correction), quantified by the electrode-specific reconstruction accuracy (see section Evaluation for details) are shown in Figure 12. As observed in the overt condition, brain areas involved in covert spectrotemporal decoding were also concentrated around STG, pre-, and post-central gyri.

**Figure 13:**
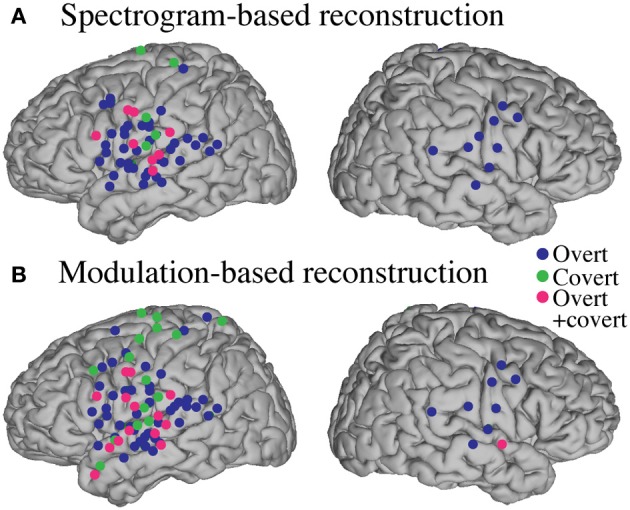
**Region of interest analysis of significant electrodes**. Significant electrodes (either overt, covert or both; *p* < 0.05; Bonferroni correction) in STG, Pre- and Post-central gyrus across subjects, co-registered with the Talairach brain template (Lancaster et al., 2000), for the spectrogram-based **(A)** and the modulation-based **(B)** reconstruction.

Anatomical differences between overt and covert informative areas were assessed for significant electrodes (either overt, covert, or both conditions; *p* < 0.05; Bonferroni correction), using an unbalanced Two-Way ANOVA, with experimental condition (overt and covert speech) and anatomical region (superior temporal gyrus, pre- and post-central gyrus) as factors. Figure 13 shows significant electrodes across subject, co-registered with the Talairach brain template (Lancaster et al., 2000). The main effect of experimental condition was significant for the spectrogram-based [*F*_(1, 116)_ = 19.6, *p* < 10^−6^] and modulation-based reconstructions [*F*_(1, 156)_ = 16.9, *p* < 10^−4^], indicating that the magnitude of reconstruction accuracy for overt speech (spectrogram: mean difference with baseline (*r*) = 0.06; modulation: mean difference = 0.1) was higher than for covert speech (spectrogram: mean difference = 0.006; modulation: mean difference = 0.01) at the level of single electrodes. The main effect of anatomical region was also significant [spectrogram: *F*_(2, 116)_ = 3.22, *p* < 0.05, and modulation: *F*_(2, 156)_ = 3.4, *p* < 0.05]. However, *post-hoc t*-tests with Bonferroni correction indicated no differences in accuracy at the level of *p* = 0.05: STG (spectrogram: mean difference = 0.05; modulation: mean difference = 0.07), pre- (spectrogram: mean difference = 0.02; modulation: mean difference = 0.05), and post-central gyrus (spectrogram: mean difference = 0.02; modulation: mean difference = 0.01). The interaction between gyrus and experimental condition was significant for the modulation-based reconstruction [*F*_(2, 156)_ = 3.6, *p* < 0.05] and marginally significant for the spectrogram [*F*_(2, 116)_ = 2.92, *p* = 0.058]. In the modulation representation, the overt condition resulted in significantly higher accuracy than the covert condition for the STG (mean difference = 0.12; *p* < 10^−5^), but not for the pre-central (mean difference = 0.06; *p* > 0.05) or the post-central gyrus (mean difference = 0.02; *p* > 0.05). This suggests that STG is the cortical area where the spectrotemporal representations of overt and covert speech have the largest absolute difference in reconstruction accuracy. Understanding the differences in the neural representations of overt and covert speech within STG is therefore a key question toward improving the spectrotemporal decoding accuracy of covert speech.

## Discussion

We evaluated a method to reconstruct overt and covert speech from direct intracranial brain recordings. Our approach was first to build a neural decoding model from self-generated overt speech, and then to evaluate whether this same model could reconstruct speech features in the covert speech condition at a level of accuracy higher than expected by chance. This technique provided a quantitative comparison of the similarity between auditory perception and imagery in terms of neural representations based on acoustic frequency and modulation content. Our results indicated that auditory features of covert speech could be decoded from models trained from an overt speech condition, providing evidence of a shared neural substrate for overt and covert speech. However, comparison of reconstruction accuracy in the two conditions also revealed important differences between overt and covert speech spectrotemporal representation. The predictive power during overt speech was higher compared to covert speech and this difference was largest in STG sites consistent with previous findings of a partial overlap of the two neural representations (Huang et al., 2002; Shuster and Lemieux, 2005; Geva et al., 2011c; Pei et al., 2011). In addition, we compared the quality of the reconstructions by assessing how well they could be identified. The quality of overt speech reconstruction allowed a highly significant identification, while in the covert speech condition, the identification was only marginally significant. These results provide evidence that continuous features of covert speech can be extracted and decoded from ECoG signals, providing a basis for development of a brain-based communication method for patients with disabling neurological conditions.

Previous research demonstrated that continuous spectrotemporal features of auditory stimuli could be reconstructed using a high gamma neural-based decoder (Pasley et al., 2012). In this study, we analyzed auditory stimuli from self-generated speech as opposed to external auditory stimulation. During self-produced speech, neural activity in human auditory cortex is reported to be suppressed (Creutzfeldt et al., 1989; Flinker et al., 2010) which has been attributed to the effect of efference copy or corollary discharge sent from the motor cortex onto sensory areas (Jeannerod, 2003). Despite this effect, we observed that high gamma activity in the superior temporal gyrus, pre- and post-central gyrus during vocalization was sufficient to reliably reconstruct continuous spectrotemporal auditory features of speech.

There is accumulating evidence that imagery and perception share similar neural representations in overlapping cortical regions (Yetkin et al., 1995; Kosslyn and Thompson, 2000; Rosen et al., 2000; Palmer et al., 2001; Aziz-Zadeh et al., 2005; Geva et al., 2011c; Cichy et al., 2012). It has been proposed that an efference copy is generated from the motor cortex through motor simulation and sent to sensory cortices enabling a comparison of actual with desired movement and permitting online behavioral adjustments (Jeannerod, 2003). Similar accounts have been proposed in speech processing (Hickok, 2001; Guenther et al., 2009; Price, 2011; Tian and Poeppel, 2012). Higher order brain areas internally induce lower level sensory cortices activation, even in the absence of actual motor output (covert). The anatomical results reported here are in agreement with these models. The relationship between overt and covert speech reconstruction depended on anatomy. High gamma activity in the superior temporal gyrus, pre- and post-central gyrus provided the highest information to decode both spectrogram and modulation features of overt and covert speech. However, the predictive power for covert speech was weaker than for overt speech. This is in accordance with previous research showing that the magnitude of activation was greater in overt than in covert speech in some perisylvian regions (Palmer et al., 2001; Pei et al., 2011; Partovi et al., 2012) possibly reflecting a lower signal-to-noise ratio (SNR) for HG activity during covert speech. Future work is needed to determine the relative contributions of SNR vs. differences in the underlying neural representations to account for discrepancies between overt and covert speech reconstruction accuracy.

A key test of reconstruction accuracy is the ability to use the reconstruction to identify specific speech utterances. At the group level, using covert reconstructions, identification performance was significant, but at a weaker level (*p* = 0.032) than overt speech identification (*p* < 10^−4^). At the individual level, covert speech reconstruction in one subject (out of seven) was accurate enough to identify speech utterances better than chance level. This highlights the difficulty in applying a model derived from overt speech data to decode covert speech. This also indicates that the spectrotemporal neural mechanisms of overt and covert speech are partly different, in agreement with previous literature (Aleman, 2004; Shuster and Lemieux, 2005; Basho et al., 2007; Pei et al., 2011). Despite these difficulties, it is possible that decoding accuracy may be improved by several factors. First, a major difficulty in this approach is the alignment of covert speech reconstructions to a reference speech segment. Variability in speaking rate, pronunciation, and speech errors can result in suboptimal alignments that may be improved by better alignment algorithms or by more advanced automatic speech recognition techniques (e.g., Hidden Markov Models). Second, a better scientific understanding of the differences between overt and covert speech representations may provide insight into how the decoding model can be improved to better model covert speech neural data. For example, the current study uses a simple model that assumes the auditory representation of covert speech imagery is equivalent to that of overt speech. If systematic differences in spectrotemporal encoding can be identified during covert speech, then the spectrotemporal tuning of the decoding model can be biased to reflect these differences in order to optimize the model for covert speech data. Further investigation of the differences in overt and covert spectrotemporal neural representation offers a promising avenue for improving covert speech decoding.

### Conflict of interest statement

The authors declare that the research was conducted in the absence of any commercial or financial relationships that could be construed as a potential conflict of interest.
